# Vibrational spectroscopy reveals the initial steps of biological hydrogen evolution†
†Electronic supplementary information (ESI) available: Complementary resonance Raman and infrared spectroscopic data. See DOI: 10.1039/c6sc01098a



**DOI:** 10.1039/c6sc01098a

**Published:** 2016-07-11

**Authors:** S. Katz, J. Noth, M. Horch, H. S. Shafaat, T. Happe, P. Hildebrandt, I. Zebger

**Affiliations:** a Institut für Chemie , Technische Universitaet Berlin , Strasse des 17. Juni 135 , D-10623 Berlin , Germany . Email: marius.horch@gmx.de ; Email: ingo.zebger@tu-berlin.de; b Fakultaet für Biologie und Biotechnologie , Lehrstuhl für Biochemie der Pflanzen , AG Photobiotechnologie , Ruhr-Universitaet Bochum , Universitaetsstrasse 150 , D-44801 Bochum , Germany; c Max-Planck-Institut für Chemische Energiekonversion , Stiftstraße 34-36 , D-45470 , Muelheim an der Ruhr , Germany

## Abstract

[FeFe] hydrogenases are biocatalytic model systems for the exploitation and investigation of catalytic hydrogen evolution. Here, we used vibrational spectroscopic techniques to characterize, in detail, redox transformations of the [FeFe] and [4Fe4S] sub-sites of the catalytic centre (H-cluster) in a monomeric [FeFe] hydrogenase. Through the application of low-temperature resonance Raman spectroscopy, we discovered a novel metastable intermediate that is characterized by an oxidized [Fe^I^Fe^II^] centre and a reduced [4Fe4S]^1+^ cluster. Based on this unusual configuration, this species is assigned to the first, deprotonated H-cluster intermediate of the [FeFe] hydrogenase catalytic cycle. Providing insights into the sequence of initial reaction steps, the identification of this species represents a key finding towards the mechanistic understanding of biological hydrogen evolution.

## Abstract 2

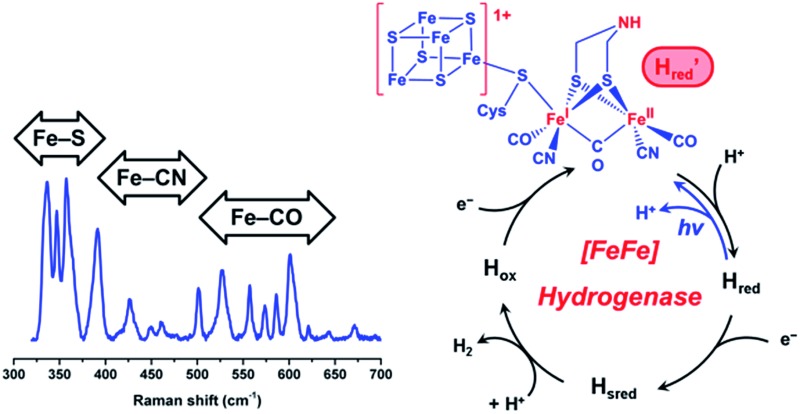
Low temperature resonance Raman spectroscopy reveals the initial, transient H-cluster intermediate during biological hydrogen production by [FeFe]-hydrogenase.

## Introduction

**Fig. 1:**
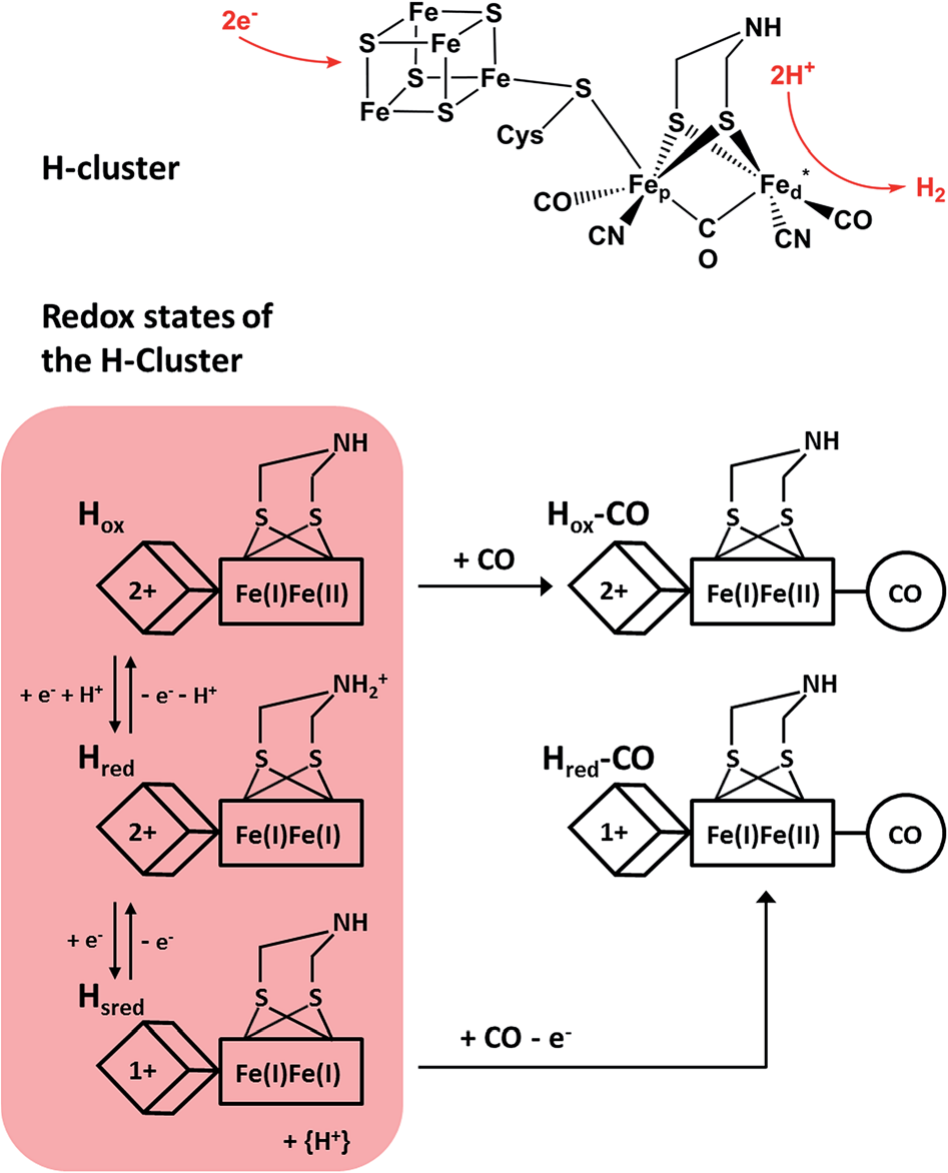
(Top) Skeletal formula representation of the H-cluster. Fe_d_ (Fe_p_) refers to the distal (proximal) iron ion of the [FeFe] sub-site, and the vacant coordination site at Fe_d_ is marked by an asterisk. Apart from the thiolate bridging the two cofactor moieties, cysteine residues are omitted for the sake of clarity. (Bottom) Schematic representations of three experimentally detected catalytic intermediates of the H-cluster (highlighted in red) are shown together with the corresponding CO-inhibited states.^5–7^ In the H_red_ state, a proton is thought to be bound to the bridging aza-dithiolate ligand, while in the H_sred_ state it is probably transferred to an amino acid side chain.^5^ Arabic and roman numbers indicate the charge of the inorganic [4Fe4S] core and the formal oxidation state of the [FeFe] moiety, respectively. For mixed valence species, the formal mono- and divalent state is arbitrarily assigned to the proximal and distal Fe atom of the [FeFe] moiety, respectively.

Molecular hydrogen represents an ideally clean fuel for future energy conversion approaches, and, thus, strategies for sustainable hydrogen cycling are of major interest. [FeFe] hydrogenases are valuable enzymes that catalyse the reversible evolution of dihydrogen by means of a catalytic centre, which is called the H-cluster (Fig. 1, top). This complex metal site consists of a ferredoxin-like [4Fe4S] cluster, which is covalently linked *via* a cysteinyl thiolate to a unique [FeFe] center.^1,2^ The two iron atoms of the [FeFe] moiety are bridged by a secondary amine dithiolate ligand,^3,4^ aza-dithiolate (adt), and coordinated by a total of two CN^–^ and three CO ligands.^5^ The distal iron atom Fe_d_ (relative to the [4Fe4S] cluster) has a vacant coordination site, which is supposed to be involved in substrate binding and conversion.

Under steady state conditions, at least three potential catalytic intermediates of the H-cluster can be clearly distinguished by infrared (IR) spectroscopy, which is able to probe the structurally sensitive CO and CN stretching vibrations of the diatomic ligands (Fig. 1, bottom).^5^ The oxidized H-cluster (H_ox_), is characterized by an [Fe^I^Fe^II^] mixed-valence ground state of the [FeFe] centre and an oxidized [4Fe4S]^2+^ cluster. The reduced H-cluster (H_red_), which exhibits an [Fe^I^Fe^I^] ground state, is obtained by adding one electron, while a further one-electron reduction yields the super reduced state (H_sred_) with an additionally reduced [4Fe4S]^1+^ cluster.^6^ Moreover, exogenous CO can be bound to the vacant coordination site of Fe_d_ in the H_sred_ and H_ox_ states, yielding H_red_–CO and H_ox_-CO, respectively.^7^ Free CO may also be released upon degradation of [FeFe] hydrogenase, and, thus, minor contributions of H_ox_-CO are typically observed for preparations of isolated enzyme.^7^ Apart from the experimentally observed redox states, additional H-cluster intermediates have been proposed to complete the catalytic cycle, whose exact sequence is still under debate. Notably, a putative isoelectronic variant of the H_red_ state, denoted as H′_red_ in the following, has been described as an oxidized [Fe^I^Fe^II^] species coupled to a reduced [4Fe4S]^1+^ cluster.^8^ While such a configuration can be expected for the first H-cluster intermediate during hydrogen evolution (*vide infra*), experimental proof is missing so far.

Resonance Raman (RR) spectroscopy is a powerful technique that provides detailed insights into selected metal–ligand vibrations and the underlying molecular coordinates of metalloproteins,^9^ *e.g.* hydrogenases.^10–16^ In the present study, we used this technique for the first time to probe the H-cluster of an [FeFe] hydrogenase under different redox conditions. Here, we chose HydA1 from the green alga *Chlamydomonas reinhardtii* as an ideal model system for spectroscopic studies, as it contains only the H-cluster and no additional cofactors that could obscure the spectra.^17^ Moreover, holo-HydA1 can be maturated *in vitro* from the ‘apo-protein’ (containing only the [4Fe4S] cluster) by addition of synthetic [FeFe] complexes.^18^ This allows for the characterization of both native and non-native H-cluster derivatives, and the corresponding cofactor building blocks can be probed separately. In the present work, we characterized these precursor forms and *in vitro*-maturated holo-HydA1 (ref. 18) by RR spectroscopy. Based on these studies, we provide insights into both H-cluster sub-sites as well as their interaction and relate the results to the catalytic mechanism of [FeFe] hydrogenase.

## Experimental details

### Heterologous expression and purification of apo-HydA1

IscR-deficient *Escherichia coli* strain BL21 (DE3) ΔiscR was used for heterologous expression of the [FeFe] hydrogenase apo-HydA1, only containing the [4Fe4S] cubane cluster, as described previously.^18–20^ The hydrogenase was purified anaerobically *via* HIS_6_-Tag IMAC,^21^ and main elution fractions were concentrated to 2 mM.

### *In vitro* maturation of purified apo-HydA1 and accumulation of defined H-cluster redox states

Holo-HydA1(adt) and its catalytically inactive analog holo-HydA1(pdt) were prepared by *in vitro* incorporation of the corresponding [FeFe]-adt and [FeFe]-pdt (propane-dithiolate bridged) complexes, at 10-fold molar excess, into apo-HydA1, according to the procedure described by Esselborn and co-workers in 2013.^18^ After desalting, the as-isolated enzymes were stored at a concentration of 2.2 mM in 0.1 M Tris–HCl buffer, pH 8, with 2 mM sodium dithionite (NaDT) to avoid damage of the enzymes by oxygen.^6,18^ Starting from this preparation, different redox states of the active site H-cluster of holo-HydA1(adt) were enriched by flushing the samples with molecular hydrogen (super-reduced state) and carbon monoxide (CO-inhibited state) for 30 min or by adding thionine in a 2-fold molar excess (oxidized state). Oxidation of as-isolated holo-HydA1(pdt) and apo-HydA1 was accomplished by incubation with thionine according to the procedure described for holo-HydA1(adt). Protein samples were stored at 193 K until further characterization.

### Resonance Raman measurements

HydA1 samples (2.2 mM) were pipetted on quartz plates and frozen in liquid nitrogen inside an anaerobic glove box. RR spectra were recorded using a confocal Raman spectrometer (LabRam HR-800, Jobin Yvon, Horiba Scientific) coupled to a liquid nitrogen-cooled CCD camera. The excitation laser beam (Ar ion laser, coherent; *λ* = 458, 488, and 514 nm) was focused onto the sample surface using a Nikon 20× objective. The laser power at the sample surface was set to 1 mW. During the measurements, samples were kept at 80 K under anaerobic conditions by a Linkam THMS600 cryostat.

For a proper comparison of RR data recorded from different HydA1 samples, spectra depicted in Fig. 3 of the manuscript were normalized as described in the following. Using the RR spectrum of thionine-oxidized holo-HydA1(adt) as a reference (‘ox’, Fig. 3A), difference spectra *Δ* = *f* × *s* – ox (black traces in Fig. 3B–D) were calculated for all other spectra *s* (corresponding to coloured traces in Fig. 3B–D) prior to baseline correction. In each case, the scaling factor *f* was adjusted in such way that the corresponding difference spectrum reflected qualitative differences between *s* and ox but not just variations of the overall intensity in the spectral region of Fe–CO/CN centred normal modes (400–700 cm^–1^). Subsequently, each baseline-corrected RR spectrum (coloured traces in Fig. 3B–D) was scaled by the same corresponding factor *f*. While this approach ensures an optimal comparability in terms of qualitative features, it might be misleading in the interpretation of relative band intensities, if the resonance enhancement for Fe–CO/CN centred normal modes varied significantly between different redox states of the [FeFe] moiety. To exclude such a scenario, we have also evaluated the band intensity of a (non-resonantly excited) phenylalanine side chain mode of the protein (at *ca.* 1005 cm^–1^, see ESI 1†). A comparison of the scaled spectra 3A–D revealed only small intensity variations with respect to this internal standard (less than 10%), thereby confirming that the above normalization procedure allows for a proper evaluation of relative band intensities.

### Infrared measurements

HydA1 samples (1.1 mM) containing 50% v/v glycerol were transferred to a gas-tight custom-made sandwich cell with CaF_2_ windows (optical path length = 55 μm) inside an anaerobic glove box. IR spectra were recorded using a Bruker IFS28 FTIR spectrometer equipped with a liquid nitrogen-cooled MCT detector. After measuring spectra at room temperature, the sample chamber was cooled to 80 K using a custom-made liquid nitrogen cryostat.^26^ Low-temperature spectra were recorded before, during, and after constant illumination using an LED panel (*λ*_max_ = 460 nm).^15^ Afterwards, the temperature was raised, and room temperature spectra were recorded again to test the integrity of all samples after measurements (see ESI 2†).

## Results and discussion

**Fig. 2:**
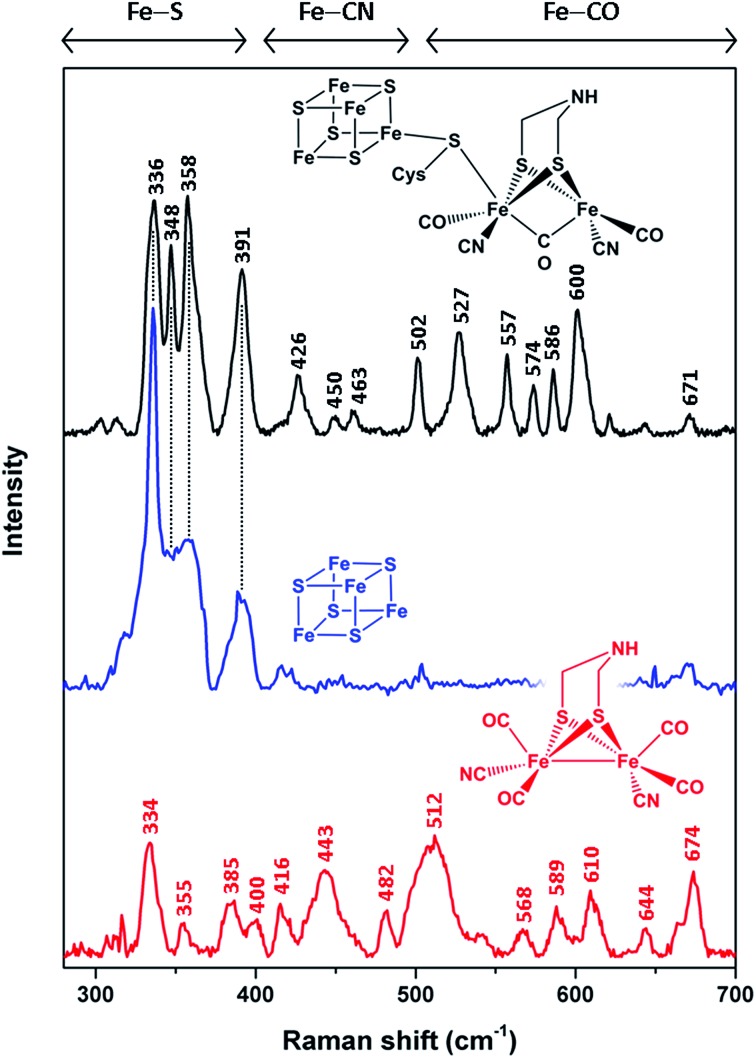
Low-temperature RR spectra (80 K) of the reduced synthetic [FeFe]-adt complex (red, 25 mM, 514 nm excitation), thionine-oxidized apo-HydA1 (blue, 1.4 mM, 458 nm excitation), and *in vitro*-matured holo-HydA1(adt) (black, 2 mM, 488 nm excitation). Color-coded schematic representations depict the chemical (cofactor) species reflected by the individual RR spectra. Apart from a thiolate bridging the two H-cluster moieties, cysteine residues are omitted for the sake of clarity. Spectral regions reflecting normal modes with major contributions from Fe–S, Fe–CN, and Fe–CO coordinates are indicated.^11–16,22–25^ Spectra of holo- and apo-HydA1 were normalized with respect to the band intensity of a (non-resonantly excited) phenylalanine sidechain mode of the protein matrix at *ca.* 1005 cm^–1^ (not shown here, see ESI 1†). The spectrum of the synthetic [FeFe]-adt complex was scaled to match the spectrum of holo-HydA1(adt) in terms of maximum band intensities in the region of Fe–CO centred normal modes.

In order to unravel the spectral marker regions, we first compare RR signatures of the synthetic [FeFe]-adt complex, the [4Fe4S] cluster-containing apo-protein, and *in vitro*-maturated holo-HydA1(adt) (Fig. 2). Bands in the region between 300 and 400 cm^–1^ are expected to originate mainly from Fe–S modes. For holo-HydA1(adt), comparison with RR spectra of prototypic cubane clusters^11–14,22,23^ and the ‘apo-protein’ indicates that these bands are largely due to the [4Fe4S] moiety, while contributions from the [FeFe] sub-site are likely to be small. Bands at higher frequencies (400–700 cm^–1^) can be clearly assigned to the [FeFe] moiety. According to (computationally supported) spectroscopic studies on [FeFe] and [NiFe] hydrogenases, bands found between 400 and 500 cm^–1^ are dominated by Fe–CN coordinates, while modes with predominant Fe–CO character are observed above 500 cm^–1^.^15,16,24,25^ Notably, the RR spectrum of holo-HydA1(adt) does not represent a simple sum of the spectra of the [FeFe]-adt complex and the [4Fe4S] centre-containing apo-protein. In line with previous observations,^4,18,27^ this indicates that geometry and electronic structure of the H-cluster are considerably modulated by the protein environment and the interactions between the two sub-sites. This effect is particularly evident for the [FeFe]-adt complex, which displays distinct changes of normal mode frequencies, band widths, and relative intensities upon incorporation into the ‘apo-protein’. These changes can be largely ascribed to interactions with the protein matrix, which imposes constraints on the cofactor structure required for its function in catalytic proton reduction. The same explanation may hold for the two sharp bands of the [4Fe4S] cluster in holo-HydA1(adt), at 348 and 358 cm^–1^, which are unresolved in the spectrum of the ‘apo-protein’.

**Fig. 3:**
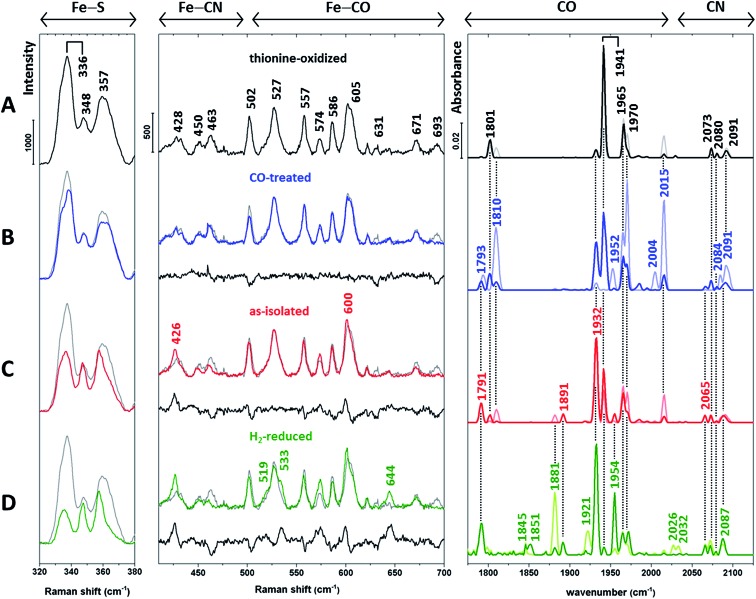
Baseline-corrected vibrational spectra of (A) thionine-oxidized, (B) CO-treated, (C) as-isolated (dithionite-reduced), and (D) H_2_-reduced *in vitro*-matured holo-HydA1(adt). Low-temperature RR spectra (80 K, 488 nm excitation) are presented in two parts for the sake of clarity. The middle panel displays the spectral region reflecting Fe–CO/CN vibrations of the [FeFe] moiety, while the left panel is dominated by normal modes of the [4Fe4S] cluster.^11–16,22–25,28^ The difference spectra in the middle panel (traces B–D, black lines) were calculated by subtracting the spectrum of thionine-oxidized holo-HydA1 (trace A, shown in grey) from coloured traces B–D prior to baseline-correction. RR spectra are normalized as described in the Experimental details section. Low-temperature IR spectra (80 K) of the CO and CN stretching modes of the [FeFe] moiety are depicted in the right panel (see Table 1 for band assignments). Lines in light and saturated colours represent spectra recorded in the dark and during blue light illumination (460 nm), respectively. Interestingly, IR spectra of H_2_-treated holo-HydA1(adt) exhibit a significant photo-induced decrease of the band at 1881 cm^–1^ and a concomitant absorbance increase at 1954 cm^–1^ (trace D). Previously, both bands were assigned to a single H_sred_ state,^6^ which appears unlikely according to this observation.

Next, we recorded RR spectra of *in vitro*-matured holo-HydA1(adt) preparations enriched in distinct H-cluster redox states (Fig. 3, left and middle). Spectra were obtained with 488 nm excitation, which provides resonance enhancement for both metal sites. The contribution of individual redox states was assessed by complementary IR measurements (Fig. 3, right) performed at the same temperature (80 K). IR spectra were recorded in the dark (lines in light colours) and during illumination with blue light (460 nm, lines in saturated colours), the latter mimicking the conditions of the RR measurements.

**Table 1:** Frequencies of IR-detectable CO and CN stretching modes previously assigned to different redox states of the HydA1 H-cluster.^6,7^ The listed frequencies correspond to measurements at 80 K and, thus, differ slightly from those reported in the above references (in parentheses). Note that bands assigned to a single H_sred_^7^ state may actually reflect two different (sub-)species, see Fig. 3D, right

Redox state	Wavenumber/cm^–1^
H_ox_	1801, 1941, 1965, 2073, 2091, (1800, 1940, 1964, 2072, 2088)
H_red_	1791, 1891, 1932, 2073, (1793, 1891, 1935, 2070, 2083)
H_sred_	1881, 1921, 1954, 2026, 2073, (1882, 1919, 1954, 2026, 2070)
H_ox_-CO	1810, 1965, 1970, 2015, 2084, 2091, (1810, 1964, 1972, 2013, 2084, 2092)
H_red_-CO	1793, 1952, 2004, 2077, (1793, 1951, 1967, 2002, 2075, 2086)

HydA1 is highly sensitive towards oxygen^29,30^ and, thus, isolated anaerobically and stored in the presence of dithionite under reducing conditions (called as-isolated in the following, Fig. 3C). Incubation of as-isolated enzyme with thionine (Fig. 3A) and hydrogen (Fig. 3D) enables the enrichment of oxidized and (super) reduced enzyme, respectively.^6^ In addition, spectral data were obtained from the CO-treated as-isolated enzyme (Fig. 3B) to identify possible contributions from CO-inhibited states.

Apart from H_sred_ and, probably, H_red_-CO, typical redox states of the H-cluster are expected to exclusively differ in the (electronic) structure of the [FeFe] moiety.^5–7^ Therefore, we first inspected IR signatures and the Fe–CO/CN region of the RR spectra (Fig. 3, middle and right). The IR spectrum of the thionine-treated sample with dominant bands at 1801, 1941, and 1965 cm^–1^ indicates an almost pure H_ox_ state (Fig. 3A, right). Therefore, the RR spectrum of this sample (Fig. 3A, left and middle) is taken as a reference and depicted in the background of all other RR spectra (Fig. 3B–D, grey lines). For the Fe–CO/CN region, differences between each spectrum and this reference are additionally highlighted by a difference spectrum shown below each trace (black line).

The IR spectra of the CO-treated sample (Fig. 3B, right) confirm the previous finding that exogenous CO is photo-dissociated from the [FeFe] moiety at low temperatures,^31^ even upon LED irradiation (460 nm). Considering the distinctly higher photon irradiance of the Raman probe laser, any contribution of CO-inhibited states to the corresponding RR spectrum (Fig. 3B, middle) can be ruled out. As shown by the IR spectra, CO-inhibited holo-HydA1(adt) (1810, 1970, and 2015 cm^–1^) observed in the dark sample (light blue line) is photo-converted to H_ox_ and H_red_, reflected by bands at 1941 and 1932 cm^–1^, respectively (dark blue line). Despite this mixture of states, the corresponding RR spectrum in the Fe–CO/CN region is nearly identical to that of the oxidized sample, as also shown by the difference spectrum (CO-inhibited – H_ox_) in Fig. 3B, middle. In the IR spectrum of as-isolated holo-HydA1(adt) (Fig. 3C, right), a mixture of H_ox_, H_red_, and H_sred_ is observed, as indicated by the characteristic marker bands discussed above and additional new bands at 1881 and 1954 cm^–1^, assigned to H_sred_. The corresponding RR spectrum differs slightly from that of thionine-treated holo-HydA1(adt). This observation is highlighted by the difference spectrum (as-isolated – H_ox_), which reflects a depopulation of H_ox_ (negative bands) in favour of H_red_ and, possibly, H_sred_ (positive bands). Incubation with H_2_ enhances these tendencies, and three new positive bands at 519, 533, and 644 cm^–1^ likely reflect the formation of H_sred_ or a related photoproduct (Fig. 3D, middle).

Thus, substantial changes in the IR spectra (Fig. 3, right), reflecting the various redox state distributions in the differently treated samples, are contrasted by rather small alterations in the respective RR spectra of the [FeFe] moiety (Fig. 3, middle). To resolve this discrepancy, we turn our attention to the RR spectral region characteristic of the [4Fe4S] cluster (Fig. 3, left). Bands in this region can be largely considered as markers for the oxidized [4Fe4S]^2+^ state, as little or no resonance enhancement is expected for the reduced [4Fe4S]^1+^ state (see ESI 1†).^32^ In particular, the most prominent band of the oxidized sample (Fig. 3A, left) at 336 cm^–1^ is a typical marker for oxidized [4Fe4S]^2+^ clusters.^22^

Apart from the H_sred_ state, which is enriched only under strongly reducing conditions (Fig. 3D, right), the [4Fe4S] sub-centre is proposed to remain in the [4Fe4S]^2+^ state for all major catalytic intermediates of the H-cluster.^6^ ¶
¶There have been reports on possible hydrogen adducts exhibiting a reduced [4Fe4S] cluster.^8,33^ However, according to the IR data, the presence of these putative species in our samples is unlikely. However, inspection of the RR spectra in the left panel of Fig. 3 reveals a gradual reduction-dependent intensity drop for the associated signals. This behaviour is best illustrated by a clear decrease of the marker band at 336 cm^–1^, indicating increasing amounts of the [4Fe4S]^1+^ state relative to the fully oxidized sample. This trend correlates with the overall enrichment of reduced holo-HydA1(adt) as inferred from the corresponding IR spectra (Fig. 3, right). Considering the unexpectedly small changes in the Fe–CO/CN region of the RR spectra (Fig. 3, middle), we therefore conclude that the [4Fe4S] cluster rather than the [FeFe] moiety is preferentially reduced under the conditions of the RR experiments, *i.e.* under laser illumination at low temperature.

These findings imply an intramolecular, photo-inducible redox reaction between the two sub-sites, which converts H_red_ (left) to a novel species, H′_red_ (right):

1 [Fe^I^Fe^I^] + [4Fe4S]^2+^ ⇌ [Fe^I^Fe^II^] + [4Fe4S]^1+^

This transformation was not observed upon LED illumination (Fig. 3, right), indicating a small absorption cross section of the underlying electronic transition and/or a low quantum yield. Thus, the high photon irradiance of the Raman probe laser‖
‖Note that the higher photon irradiance (compared to LED illumination) is due to the small spot size rather than the absolute power of the laser. Thus, these conditions cannot be easily reproduced in IR measurements that probe a much larger sample volume. is necessary for the photoreaction to proceed.

In line with the IR data (Fig. S3† and 3D, right), the redox equilibrium of eqn (1) is typically towards the left-hand side, implying a higher reduction potential for the [FeFe] moiety than for the [4Fe4S] sub-site. From a thermodynamic point of view, this equilibrium is not expected to be reversed at low temperatures, indicating that H′_red_ is kinetically stabilized under these conditions by a hindrance of the back-reaction. Electron tunnelling itself is not temperature-dependent, and the overall rate of efficient electron transfer reactions (*vide infra*) is not expected to vanish at low temperatures, even if nuclear quantum effects are neglected.^34^ Thus, a photo-induced electron transfer from the [FeFe] to the [4Fe4S] sub-site alone is insufficient to explain this phenomenon. We therefore conclude that the conversion of H_red_ involves an additional elementary step, which enables the kinetic stabilization of H′_red_ during the RR measurements. This step is proposed to be a proton transfer to a nearby base (most likely cysteine C169),^35^ in line with photoreactions of catalytic intermediates in [NiFe] hydrogenases.^15^ The bridging adt ligand of the [FeFe] moiety is the most likely proton donor, since it is supposed to be protonated in H_red_ but not in H_ox_.^5^ Considering the similar [FeFe] RR signatures of H_ox_ and H′_red_ (Fig. 3, middle), we therefore conclude that the adt ligand is deprotonated in the latter species as well. Thus, the overall reaction reads as

2 C169–S^–^ + [Fe^I^Fe^I^]–NH_2_^+^ + [4Fe4S]^2+^ ⇌ C169–SH + [Fe^I^Fe^II^]–NH + [4Fe4S]^1+^

In line with studies on functional [FeFe] mimics,^36^ this finding also suggests that the different protonation states of the adt ligand may stabilize the isoelectronic variants H_red_ and H′_red_ through charge compensation at the [FeFe] moiety.

To validate the structure of H′_red_, we next aimed at investigating the impact of proton donor and acceptor sites on its formation. C169 has been previously reported to be involved in proton transfer from or towards the [FeFe] moiety,^35^ and a C169S derivative of native HydA1 was found to have no or very low catalytic activity.^33,37^ This species appeared to be trapped in the inactive H_trans_ state,^37^ which is presumably characterized by an [Fe^II^Fe^II^], [4Fe4S]^1+^ ground state and a hydroxo ligand at Fe_d_.^38–41^ Similar to the H_red_/H′_red_ transformation, conversion of H_trans_ to H_ox_ would require intramolecular electron transfer between the [4Fe4S] and [FeFe] moieties. In HydA1 C169S, this process may be thermodynamically disfavoured due to the impossibility to provide a proton for water removal and charge compensation at the [FeFe] moiety. While this situation excludes insights into H′_red_, it indicates that electron transfer between the H-cluster sub-sites may indeed necessitate proton translocation, as proposed for the H_red_/H′_red_ transformation.

In addition to mutagenesis, the introduction of non-native [FeFe] species into the HydA1 H-cluster provides an alternative means to study individual reaction steps in detail.^4,18^ In particular, replacement of adt by propane-dithiolate (pdt) disables proton transfer steps involving the bridging ligand, thereby providing further insights into these processes. Oxidized holo-HydA1(pdt) exhibits the H_ox_ state, while the reduced form has been described as an [Fe^I^Fe^II^], [4Fe4S]^1+^ species.^7^ These observations support the need of a protonated bridging ligand for stabilizing the [Fe^I^Fe^I^], [4Fe4S]^2+^ ground state of H_red_ and furthermore indicate that reduced holo-HydA1(pdt) represents a thermodynamically stable model for the proposed H′_red_ state of native holo-HydA1(adt). In line with previous IR studies and the electronic ground states proposed therein (*vide supra*),^7^ we find that the [FeFe] RR signatures of oxidized and reduced holo-HydA1(pdt) are very similar, while a major intensity decrease is observed for the [4Fe4S] cluster signals upon reduction (Fig. S2†). This observation agrees with the data obtained for H′_red_ (Fig. 3). In addition, overall RR spectroscopic signatures of holo-HydA1(pdt) and holo-HydA1(adt) are very similar, both in the oxidized and reduced state (Fig. S2†). Thus, we conclude that H′_red_ and reduced holo-HydA1(pdt) can be indeed described by the proposed [Fe^I^Fe^II^], [4Fe4S]^1+^ configuration.

**Fig. 4:**
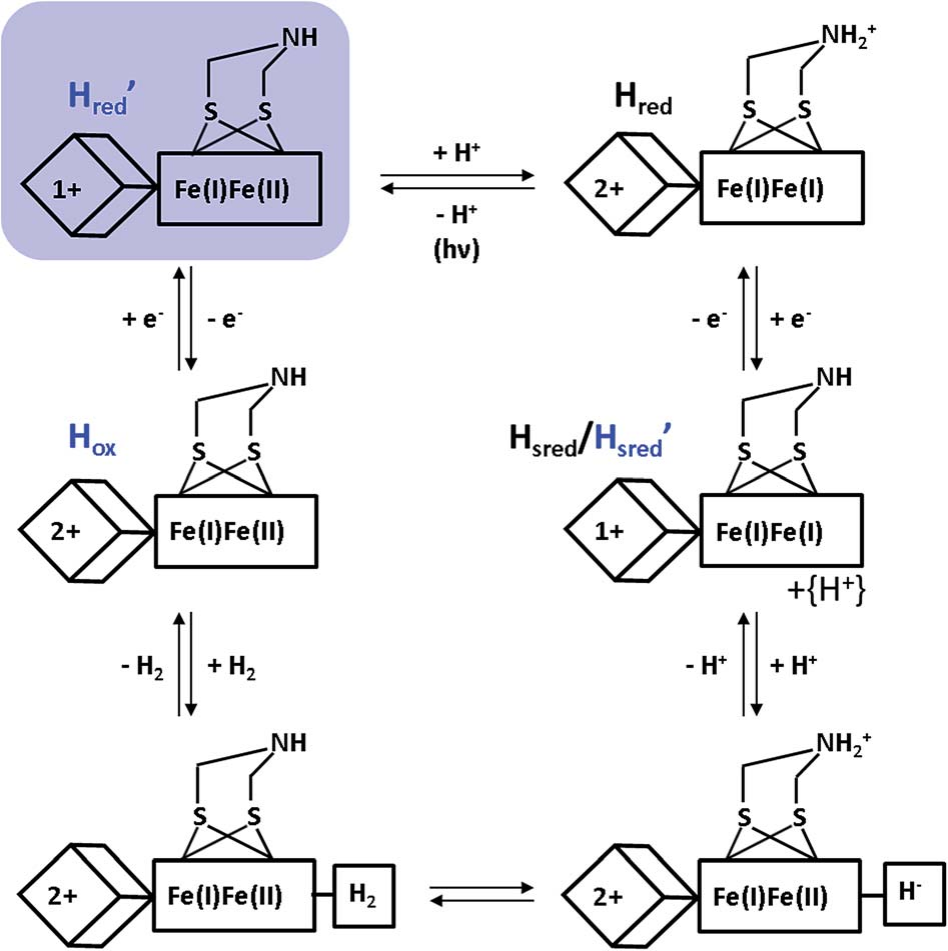
New proposal for the catalytic cycle of [FeFe] hydrogenase, expanded from ref. 5. Intermediates probed by RR spectroscopy are labelled in blue, and the novel H′_red_ species is additionally highlighted by a light blue shade. Arabic and roman numbers indicate the charge of the inorganic [4Fe4S] core and the formal oxidation state of the [FeFe] moiety, respectively. For mixed valence species, the formal mono- and divalent state is arbitrarily assigned to the proximal and distal Fe atom of the [FeFe] moiety, respectively.

Despite the special requirements for the enrichment of the H′_red_ species in the RR experiment, this intermediate may plausibly contribute to the catalytic cycle (Fig. 4). Notably, a thermal equilibrium according to eqn (1) has been previously suggested,^8^ but the right-hand side configuration H′_red_ has not been observed experimentally prior to this study. Based on our findings, we propose that this species represents the first H-cluster intermediate in hydrogen evolution, which is formed by one-electron reduction of H_ox_ and rapidly transformed to H_red_ *via* an intramolecular electron and proton transfer to the [FeFe] sub-site (Fig. 4). This conclusion is consistent with the apparently lower reduction potential of the [4Fe4S] site (*vide supra*) and its direct redox interaction with external electron donors of HydA1 (Fig. 1, top). Moreover, the thermodynamic instability of H′_red_ *versus* H_red_ may hinder reverse electron transfer from the [FeFe] moiety *via* the [4Fe4S] centre towards an external electron acceptor. This argumentation is in line with the enzyme's bias towards hydrogen evolution,^42^ supporting the previous assumption that lower reduction potentials of (distal) [4Fe4S] clusters favour proton reduction in hydrogenases.^43^

## Conclusions

In conclusion, this work provides detailed insights into RR-detectable metal–ligand modes of the entire H-cluster and its cofactor building blocks. Using these vibrational markers as sensitive probes for the redox states of the [FeFe] and [4Fe4S] sub-sites, a novel intermediate of the H-cluster, H′_red_, was discovered. In line with data from a non-native H-cluster derivative, this metastable species is proposed to be characterized by an unusual [Fe^I^Fe^II^], [4Fe4S]^1+^ ground state and a deprotonated adt ligand, which identifies H′_red_ as the missing first H-cluster intermediate during biological hydrogen production by [FeFe] hydrogenases. This finding represents the first detection of a transient intermediate of these enzymes that is inaccessible under steady state conditions. In a wider sense, the present study highlights the capability of low-temperature spectroscopy to probe and characterize otherwise inaccessible intermediates in hydrogenase and related metalloenzymes.
