# Supplementary material for: Vibrational spectroscopy reveals the initial steps of biological hydrogen evolution
Source: Chem Sci. 2016 Jul 11;7(11):6746–52. doi: 10.1039/c6sc01098a (PMC5355867; doi:10.1039/c6sc01098a)
Supplement: Supplementary file 1 [file SC-007-C6SC01098A-s001.pdf]

Electronic Supplementary Material (ESI) for Chemical Science.
This journal is © The Royal Society of Chemistry 2016

Supplementary Information

Vibrational Spectroscopy Reveals the Initial Steps of Biological Hydrogen Evolution

S. Katz,[a#] J. Noth,[b#] M. Horch,[a*] H.S. Shafaat,[c‡] T. Happe,[b] P. Hildebrandt,[a] I. Zebger[a*]

[a]Institut für Chemie, Technische Universitaet Berlin, Strasse des 17. Juni 135, D-10623 Berlin, Germany.

[b]Fakultaet für Biologie und Biotechnologie, Lehrstuhl für Biochemie der Pflanzen, AG Photobiotechnologie, Ruhr-Universitaet Bochum, Universitaetsstrasse 150, D-44801 Bochum, Germany.

[c]Max-Planck-Institut für Chemische Energiekonversion, Stiftstraße 34-36, D-45470, Muelheim an der Ruhr, Germany.

[‡]Present address: Department of Chemistry and Biochemistry, 100 W. 18th Ave, Columbus, OH 43210, USA

[#]These authors contributed equally.

[*]email: marius.horch@gmx.de

ingo.zebger@tu-berlin.de

Contents

SI 1: Complementary Resonance Raman Spectroscopic Data (p. S2)

SI 2: Complementary Infrared Spectroscopic Data (p. S5)

References (p. S7)

SI 1: Complementary Resonance Raman Spectroscopic Data

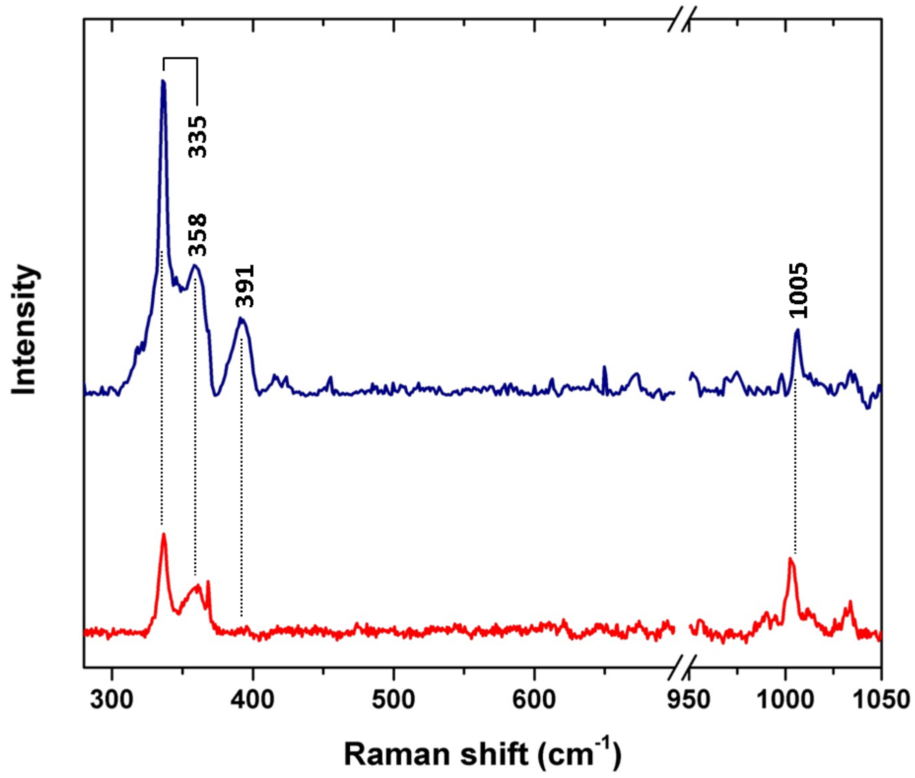

Figure S1: Baseline-corrected low-temperature (80K) resonance Raman spectra of apo-HydA1 in the as-isolated, dithionite-reduced (red) and thionine-oxidized (blue) state (458 nm excitation). Signals arising from an excess of the oxidizing thionine agent were subtracted from the spectrum of oxidized apo-HydA1. Spectra were normalized with respect to the band intensity of a (non-resonant) phenylalanine side chain mode of the protein matrix at ca. 1005 cm^{-1}.

Resonance Raman (RR) spectra of apo-HydA1 in the as-isolated (dithionite-reduced) as well as thionine-oxidized state were recorded in order to assess contributions from the reduced and oxidized [4Fe4S] cluster, respectively (Fig. S1). Both spectra exhibit very similar frequencies and relative band intensities, but absolute intensities are much higher for oxidized apo-HydA1 (blue trace) than for the reduced preparation (red trace). Thus, the weak bands in the RR spectrum of the reduced sample are attributed to residual contributions of the oxidized form. Since no additional bands can be observed, the reduced [4Fe4S] cluster has to be considered as RR-silent. This finding confirms that [4Fe4S] signals of different apo-HydA1 preparations can be entirely assigned to varying contributions of the oxidized state.

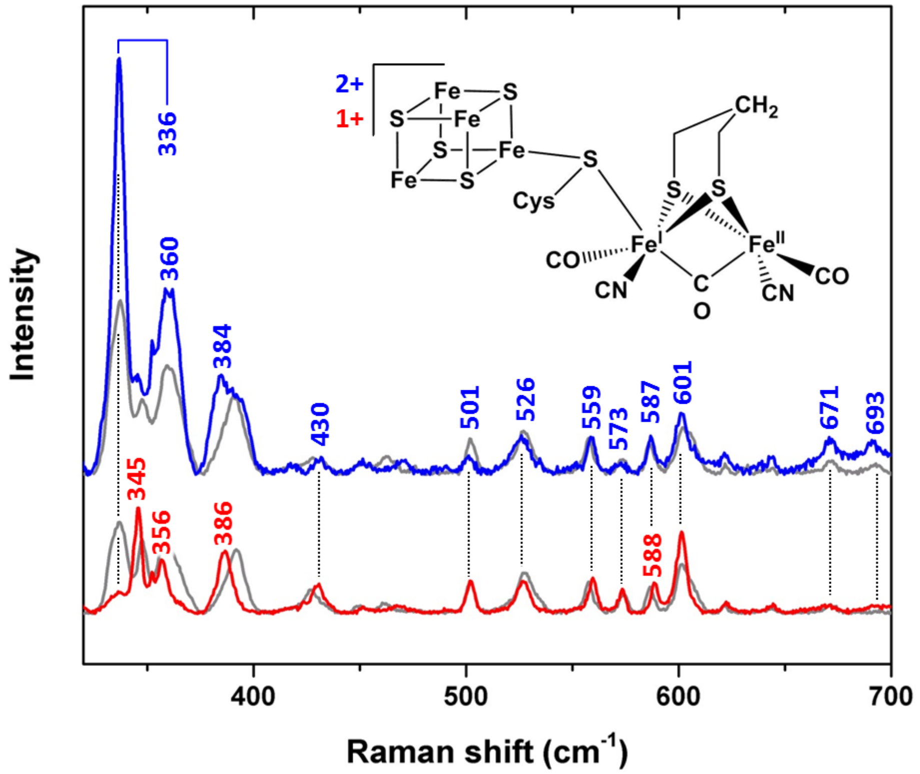

Figure S2: Baseline-corrected low-temperature (80K) resonance Raman spectra of holo-HydA1(pdt) in the as-isolated, dithionite-reduced (red) and thionine-oxidized (blue) state (488 nm excitation). The corresponding spectra of holo-HydA1(adt) are shown as grey traces in the background (see also Figure 3). The structure of holo-HydA1(pdt) is depicted schematically, and differences between the oxidized and reduced forms are colour-coded. Spectra of holo-HydA1(adt) were normalized as described in the Experimental Details section of the manuscript, while those of holo-HydA1(pdt) were scaled to match the former in terms of approximate band intensities in the region of Fe–CO/CN centred normal modes (400 – 700 cm^{-1}).

In Fig. S2, RR spectra of oxidized and reduced holo-HydA1(pdt) and holo-HydA1(adt) are compared. For both enzyme species, reduction results in a major intensity decrease of signals corresponding to Fe–S related normal modes (below 400 cm^{-1}). This indicates that the additional electron is located at the [4Fe4S] moiety rather than the [FeFe] sub-site under the experimental conditions of the RR measurements, confirming the proposed [$Fe^{I}Fe^{II}$], $[4Fe4S]^{1+}$ ground state for both cases. Moreover, RR spectra of holo-HydA1(pdt) and holo-HydA1(adt) are very similar, both in the oxidized and reduced state, which gives further evidence for the proposed structural similarities of the probed species.

Nonetheless, RR spectra of both enzymes are not completely identical. In the oxidized state, band intensities of [4Fe4S] signals of holo-HydA1(pdt) are clearly higher than those of holo-HydA1(adt) if spectra are scaled with respect to the Fe–CO/CN region. In line with band intensities of a (non-resonant) phenylalanine side chain mode of the protein matrix (not shown here, see Fig. S1), this can be explained by a higher amount of apo-HydA1 in the holo-HydA1(pdt) sample. Contrarily, [4Fe4S] signals of reduced holo-HydA1(pdt) are even lower than those observed for holo-HydA1(adt) under comparable conditions, indicating a higher amount of the reduced $[4Fe4S]^{1+}$ state. This observation is in line with the fact that holo-HydA1(pdt) is completely reduced in the as-isolated (dithionate-reduced) state, while holo-HydA1(adt) exhibits a mixture of species including considerable amounts of the oxidized H_{ox} state (see Figure 3C, right).[1,2] Consistently, the reduction potential of the single redox transition of holo-HydA1(pdt) was reported to be higher than that of the H_{ox}/H_{red} couple of holo-HydA1(adt).[3]

SI 2: Complementary Infrared Spectroscopic Data

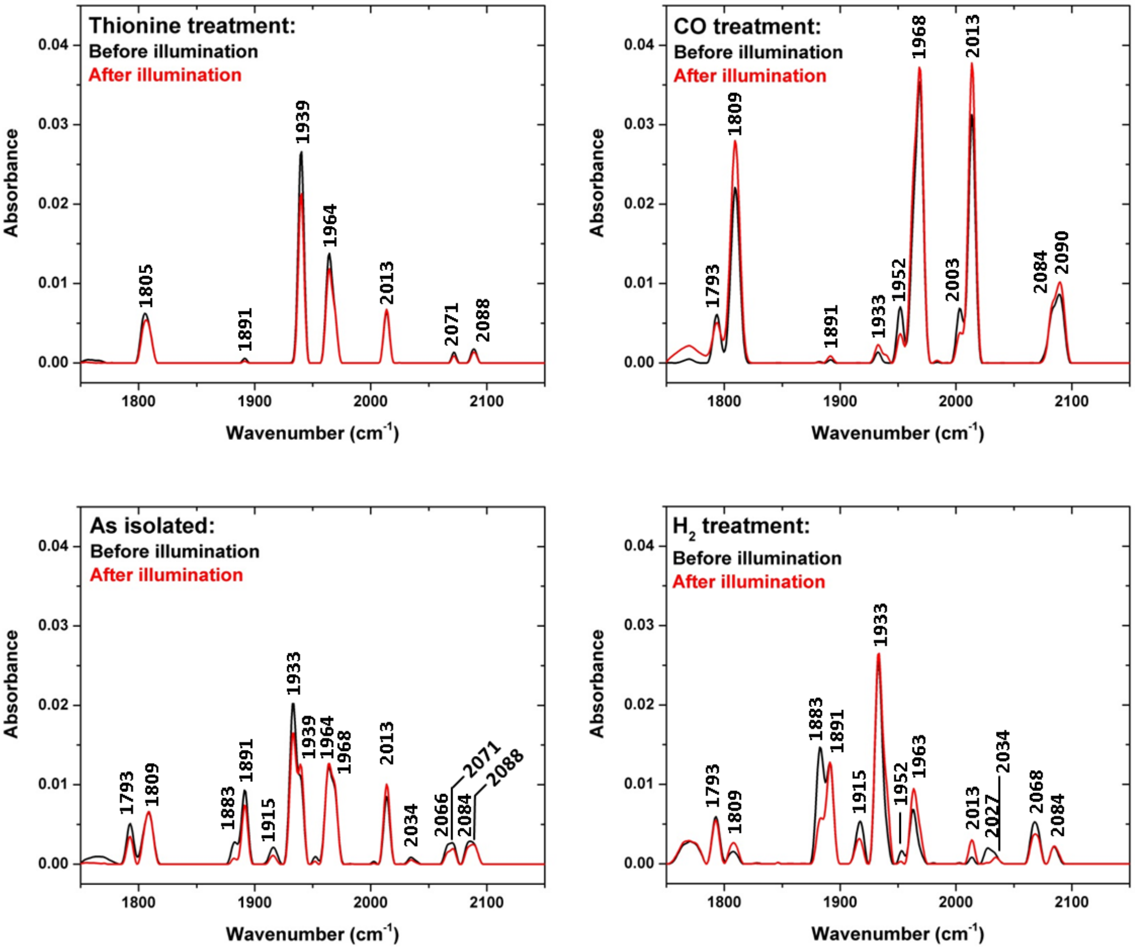

Figure S3: Room-temperature infrared spectra of holo-HydA1(adt) recorded in the dark before (black) and after (red) blue light illumination (460 nm) at 80K. Samples were treated as indicated (*vide supra*).

Baseline-corrected room-temperature infrared (IR) spectra of holo-HydA1(adt) recorded in the dark before (black) and after (red) blue light illumination (460 nm) at 80 K are shown in Fig. S3. IR spectra of the thionine-treated sample are qualitatively identical, indicating reversibility of all processes (upper left). In the CO-treated sample, a slight conversion from H_{red}-CO to H_{ox}-CO during measurements can be inferred from decreased band intensities at 1952 and 2003 cm^{-1} and an absorbance increase at 1809 and 2013 cm^{-1} (upper right, red *vs.* black spectrum). In the as-isolated and hydrogen-treated samples (bottom left and right), some depopulation of H_{sred} and H_{red} can be observed, as indicated by an absorbance decrease at 1883 / 1952 and 1891 / 1933 cm^{-1}, respectively. These effects are explained by slight oxygen

leakage into the IR cell during the slow process (several hours) of heating the sample back to ambient temperature. This process gives rise to an oxidation and partial degradation of the samples as evident from increased band intensities at 1809 (H_{ox}-CO), 1939 (H_{ox}), 1963 (H_{ox}, H_{ox}-CO), and 2013 (H_{ox}-CO) cm^{-1}.

References

1 J. Esselborn, C. Lambertz, A. Adamska-Venkatesh, T. Simmons, G. Berggren, J. Noth, J. Siebel, A. Hemschemeier, V. Artero, E. Reijerse, M. Fontecave, W. Lubitz and T. Happe, *Nat. Chem. Biol.*, 2013, **9**, 607.

2 G. Berggren, A. Adamska, C. Lambertz, T. R. Simmons, J. Esselborn, M. Atta, S. Gambarelli, J. M. Mouesca, E. Reijerse, W. Lubitz, T. Happe, V. Artero and M. Fontecave, *Nature*, 2013, **499**, 66.

3 A. Adamska-Venkatesh, D. Krawietz, J. Siebel, K. Weber, T. Happe, E. Reijerse and W. Lubitz, *J. Am. Chem. Soc.*, 2014, **136**, 11339.
